# Household latrine utilization and its association with educational status of household heads in Ethiopia: a systematic review and meta-analysis

**DOI:** 10.1186/s12889-018-5798-6

**Published:** 2018-07-20

**Authors:** Cheru Tesema Leshargie, Animut Alebel, Ayenew Negesse, Getachew Mengistu, Amsalu Taye Wondemagegn, Henok Mulugeta, Bekele Tesfaye, Nakachew Mekonnen Alamirew, Fasil Wagnew, Yihalem Abebe Belay, Aster Ferede, Mezinew Sintayehu, Getnet Dessie, Dube Jara Boneya, Molla Yigzaw Birhanu, Getiye Dejenu Kibret

**Affiliations:** 1 Department of Public Health, College of Health Sciences, Debre Markos University, Debremarkos, Ethiopia; 2 Department of Nursing, College of Health Sciences, Debre Markos University, Debremarkos, Ethiopia; 3 Department of Human Nutrition and Food Science, College of Health Sciences, Debre Markos University, Debremarkos, Ethiopia; 4 Department of Medical Laboratory technology, College of Health Sciences, Debre Markos University, Debremarkos, Ethiopia; 5 Department of Biomedical Science, School of Medicine, Debre Markos University, Debremarkos, Ethiopia

**Keywords:** Latrine-utilizations, Educational-status, Systematic-review, Meta-analysis, Ethiopia

## Abstract

**Background:**

Ethiopia has been experiencing a high prevalence of communicable diseases, which resulted in high morbidity, mortality, and hospital admission rates. One of the highest contributing factors for this is lower level of latrine utilization. There had been significantly varying finding reports with regard to the level of latrine utilization and its association with education level from different pocket studies in the country. Therefore, this systematic review and meta-analysis was aimed to estimate the pooled prevalence of household latrine utilization and its association with education status of household heads, in Ethiopia using available studies.

**Methods:**

This systematic review and meta-analysis was conducted using available data from the international databases, including PubMed, Google Scholar, Science direct, Cochrane library and unpublished reports. All observational studies reporting the prevalence of latrine utilization in Ethiopia were included. Four authors independently extracted all necessary data using a standardized data extraction format. STATA 13 statistical software was used to analyze the data. The Cochrane Q test statistics and *I*^*2*^ test were used to assess the heterogeneity between the studies. A random effect model was computed to estimate the pooled level of latrine utilization in Ethiopia. In addition, the association between latrine utilization and the educational level of the users was analyzed.

**Results:**

After reviewing of 1608 studies, 17 studies were finally included in our meta-analysis. The result of 16 studies revealed that the pooled prevalence of latrine utilization level in Ethiopia was 50.02% (95%CI: 40.23, 59.81%). The highest level (67.4%) of latrine utilization was reported from Southern Nations Nationality and People regional state, followed by Amhara regional state (50.1%). Participants who completed their high school and above education were more likely (OR: 1.79, 95%CI: 1.05, 3.05) to utilize latrine compared to those who did not attend formal education.

**Conclusion:**

In Ethiopia, only half of the households utilize latrine and the level of utilization has significant association with educational status.

**Electronic supplementary material:**

The online version of this article (10.1186/s12889-018-5798-6) contains supplementary material, which is available to authorized users.

## Background

Communicable diseases are serious public health problems, affecting billions of people around the world, mainly the third world countries [1, 2]. Latrine utilization, the main determinant for communicable diseases control, is still at its lower level in developing countries including Ethiopia [3]. Access to safe drinking water and sanitation is a basic necessity that is vital for human health and among the basic human rights declared by the United Nations. Ensuring sanitation demands the availability of facilities and services for the safe disposal of human excreta. It is one of the components of the sustainable development goals that are set to be achieved by 2030 [4–7].

Worldwide, a tremendous progress has been made in increasing access to facilities that ensure hygienic separation of human excreta from human contact. More than half of the global population used basic sanitation services and nearly two out of five people (39%) utilized safely managed sanitation services. Nevertheless, billions of people still remained without even the basic sanitation services and around 800 million people used unimproved facilities. Most countries are moving off the track to attain the desired coverage for sanitation set in the sustainable development goals [8–10].

In communities where access to improved sanitation facilities is low, people are forced to engage in unsafe practice of open defecation. This practice continues to be a major challenge and about 2.3 billion people who still lack basic sanitation service either practice open defecation (892 million) or use unimproved facilities such as pit latrines without a slab or platform, hanging latrines or bucket latrines (856 million) [11]. In sub-Saharan Africa, the number of people who defecate in the open field rose from 204 to 220 million by 2015 [8, 9, 12, 13]. Diarrheal and other communicable diseases are often linked with poor sanitation and open defecation. Moreover, higher rates of open defecation are also associated with significant socioeconomic, environmental and major public health consequences affecting the overall health and dignity of mankind, the most vulnerable groups being women and children [1, 14–19].

Increasing availability and proper utilization of latrines is essential and a cost-effective strategy to overcome disease burden associated with improper excreta management [20–22]. The use of latrines can be affected by a range of behavioral, cultural, social, geographic and economic factors differing across communities [23–29].

According to the 2016 Ethiopian Demographic and Health Surveys report, 56% of the rural households use unimproved toilet facilities. One in every three households in the country has no toilet facility [30]. The inauguration of the health extension program in 2003 and the national water supply, sanitation and hygiene [31] program contributed much to the improved coverage of latrines across the country. However, achieving real gains in increasing latrine use and quality remained as a challenge [32–35].

In Ethiopia, different fragmented and small studies have been conducted to assess the level of latrine utilization. Nevertheless, the findings of these studies reported highly varying figures. Some of the findings showed as the level of latrine utilization is at a good progress, while some others revealed the awkward aspect. The previous studies also indicated the presence of significant variability in latrine utilization from region to region [36, 37].

Determining the pooled prevalence of latrine utilization at a country level will provide an overall figure with better estimation accuracy. Therefore, this systematic review and meta-analysis was aimed at estimating the pooled prevalence of latrine utilization and its association with education level. The findings from this study will have a paramount importance for decision makers revealing at what level the country is with regard to latrine utilization.

## Methods

### Searching strategies

This systematic review and meta-analysis was conducted to estimate the pooled prevalence of latrine utilization and its association with educational level of the user in Ethiopia. To conduct this study, all potentially relevant articles, grey literatures, and government reports were meticulously searched. The Preferred Reporting Items of Systematic Reviews and Meta-Analysis (PRISMA) checklist guideline was used to ensure the scientific rigor [38]. We searched articles from international databases including Cochrane library, PubMed, Google Scholar, and Science direct. The reviewers used the following keywords “prevalence”, “(“toilet facilities“[MeSH Terms] OR (“toilet“[All Fields] AND “facilities“[All Fields]) OR “toilet facilities“[All Fields] OR “latrine“[All Fields]) AND (“utilization“[Subheading] OR “utilization”[All Fields]) AND (“ethiopia“[MeSH Terms] OR “ethiopia”[All Fields]) to get published articles from above mentioned databases.

“The search was carried out from September to October, 2017. All articles published until October, 2017 were included in the review.

### Inclusion and exclusion criteria

The current meta-analysis and systematic review included studies conducted only in Ethiopia and that reported the level of latrine utilization, articles published in the scientific journals and grey literatures. Studies written in English language and full-text articles only were considered. In addition, the review considered all observational study designs (Cross-sectional, case-control, and cohort) reported the level of latrine utilization in Ethiopia. We excluded articles which were not able to be accessed for full article text after communicating the principal investigator of the primary studies by email at least three times.

### Outcome of interest

The primary outcome of interest was the pooled prevalence of latrine utilization. The prevalence was computed from the proportion in which the number of individuals who had proper latrine utilization to the total number of households with functional latrine multiplied by 100. Estimate of the association between educational status and level of latrine utilization was also a second outcome.

### Study setting

This systematic review and meta-analysis was conducted in Ethiopia. The country is located in the Horn of Africa with projected population of 107,421,970 by 2018 year. The country is divided into nine regions and two administrative cities. The regions are Afar, Amhara, Benishangul-Gumuz, Gambella, Harari, Oromia, Southern Nations, Nationalities, and Peoples of Ethiopia, and two city administrates are Addis Ababa and Dire Dawa [39].

#### Operational definitions

**Improved sanitation facilities (*Latrine)*** are those designed to hygienically separate excreta from human contact. These include wet sanitation technologies (flush and pour flush toilets connecting to sewers, septic tanks or pit latrines) and dry sanitation technologies (ventilated improved pit latrines; pit latrines with slabs; or composting toilets) [11].

**Latrine utilization** – households with either shared or private functional latrines functional latrines and the family disposed the faeces of under-five children in a latrine, no observable faeces in the compound, no observable fresh faeces on the inner side of the squatting hole and the presence of clear foot-path to the latrine is uncovered with grasses or other barriers of walking [1].

**Education categories**: The primary studies classified education for the head of the households as 1) not attended formal education, 2) attended primary education (1–8), 3) attended secondary Educations and 4) college and above.

### Data abstraction

Four authors (CTL, AA, AF and HM) independently searched the studies, articles, and reports, and extracted all necessary data using a standardized data extraction format using Microsoft Excel. The extracted parameters were: primary author, publication year, region where the study was conducted, the study design used, sample size, level of latrine utilization, and quality of each study. Then, three authors (AT, GDK and NM) checked the data extraction process. Finally, nine authors (AN, BT, FW, DJB, GM, YA, GDK, MYB and MS) participated in resolving the disagreement.

### Quality assessment of the studies

We used Newcastle-Ottawa Scale adapted for cross-sectional studies quality assessment to assess the quality of each study [40]. The tool has mainly three sections; the first section grades from five stars and mainly focuses on the methodological quality of each study (sample size, response rate and sampling technique). The second section deals with the comparability of the studies, with a possibility of two stars to be gained. The last section deals with the outcomes and statistical analysis of the original study with a possibility of three stars to be gained (Additional file 1). Two authors independently assessed the quality of each original study. Disagreements between two authors were resolved by taking the mean score of the two authors. Finally, researches with a scale of ≥6 out of 10 were considered as achieving high quality. This cut-off point was declared after reviewing relevant literatures.

### Data analysis

The extracted data were compiled in Microsoft Excel format and analyzed using STATA version 13 statistical software. The binomial distribution formula was used to calculate standard error for each eligible original article. Heterogeneity between studies was assessed using Cochran’s Q- statistics and Higgins’ and Thompson’s *I*^2^ test [41]. As the preliminary output of the test statistics revealed a significant heterogeneity among studies (*I*^2^ = 99.5%, *p* = 0.00), random Effects meta-analysis model was used for approximation of the Der Simonian and Laird’s pooled effect. Subgroup analysis was also performed among regions, study setting and education in relation to the latrine utilization as well as trends of latrine utilization was made. To reduce the random variations between the individual point estimates of the primary study, a subgroup analysis was carried out based on study settings (regions). Possible source of heterogeneity was also identified by Univariate Meta-regression by taking the sample size and year of publication as covariates. Furthermore, Egger and Begg tests at 5% significant level were employed to assess publication bias [42]. The point prevalence with its corresponding 95% confidence interval was presented using forest plot. In this forest plot, the size of each individual box revealed the weight of the study, while each crossed line refers to 95% confidence interval. We conducted log-odd ratio for the second outcome (the relationship between latrine utilization and educational status of the households.

## Results

**Fig. 1:**
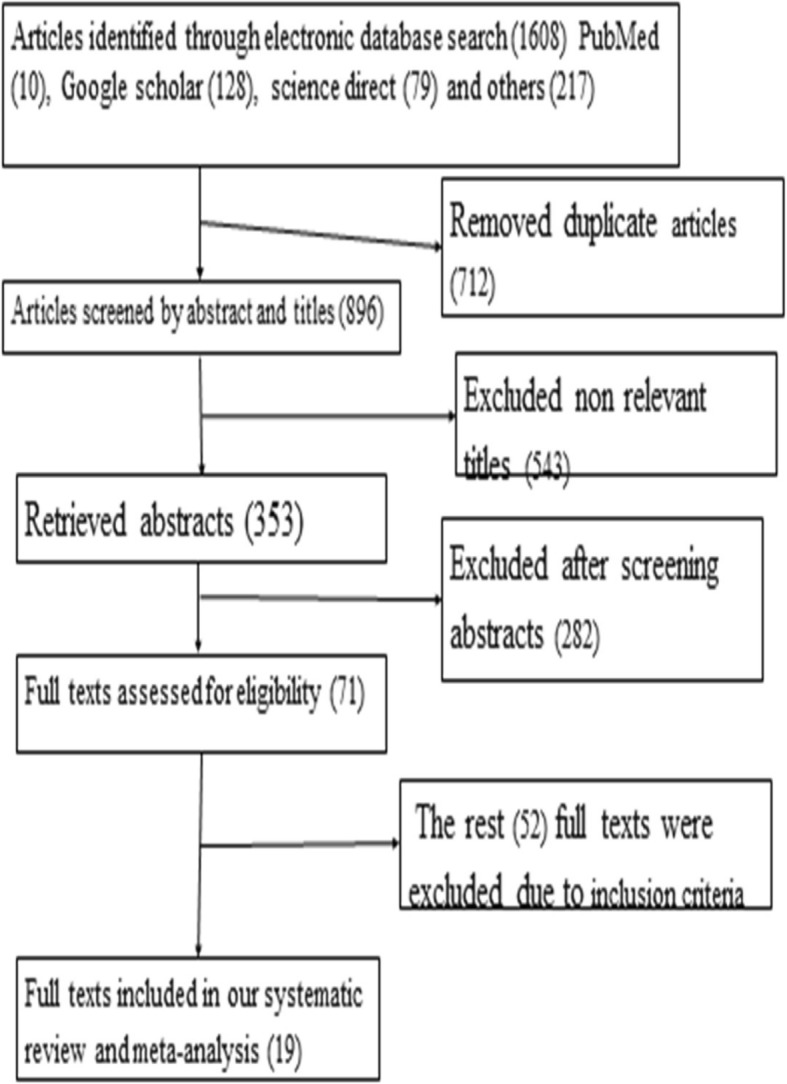
Flow chart to describe the selection of studies for a systematic review and meta-analysis of the level of latrine utilization and is association with educational status at Ethiopia

One thousand six hundred eight (1608) primary studies that addressed latrine utilization and associated factors were searched using both through PubMed, Google Scholar, science direct and grey as well as the government reports. Seven hundred twelve (42.3%) of these identified articles were excluded because of similarity and duplicated articles. Among the remaining 896articles, 543 articles were excluded after reviewing their titles for a reason of relevance for our objective. The rest 353 articles were screened for abstracts and 286 were excluded after reading their abstract sections. Therefore, 71 full-text articles were accessed and assessed for eligibility based on the pre-set criteria, and from these 52 were excluded for not fitting the inclusion criteria. Finally, 19 studies fulfilled the eligibility criteria and included in the final meta-analysis (Fig. 1).

### Overview of included studies

These 19 (of which 2 were unpublished) studies were published from 1999 to 2017. In the current meta-analysis, 966,362 study participants were involved to estimate the pooled prevalence of latrine utilization in Ethiopia in which the lowest (30.99%) latrine utilization was observed from a study conducted at Akaki, Oromia region [43] while the highest prevalence (99.4%) was reported from a study conducted in Dembia district of Amhara region [44]. Regarding the study design, all (100%) of the studies were cross-sectional study designs. The sample sizes of the studies ranged from 355 to 955,985. This meta-analysis and systematic review used data taken from primary studies of five (5) (Amhara, south nation and nationality people of Ethiopia, Oromia, Tigray and Harari) regions of Ethiopia that shares eight (42%), 4 (21%), 4 (21%), 2 (1%) and 1 (0.5%) respectively (see the Additional file 2).

### Meta-analysis

As indicated above in the Additional file 2, 19 studies were found to be eligible for the analysis. Of these, two studies [44, 45] were excluded from forest plot of the pooled level of latrine utilization after we did sensitiy analysis. The sensitivity analysis for Amhara, Tigray, SNNP and others (Oromia and Harar) regions were revealed as (I^2^ = 98.3, *p* value = 0.001), (I^2^ = 98.9, *p* value = 0.001), (I^2^ = 62.7, *p* value = 0.001) respectively. Seventeen articles were considered to determine the pooled prevalence of latrine utilization in Ethiopia that found to be 50.2% (95% CI: 40.23, 59.81%). High heterogeneity, (I^2^ = 99.5, *p* value < 0.001), was observed between 17 primary studies included in this review. As a result, to reduce it, we performed a subgroup analysis (I^2^ = 99.5, *p* value = 0.001) and come up a slight improvement. The regional subgroup analysis revealed that significant regional variation regarding latrine utilization was observed across the country. Southern nation nationalities and people of Ethiopian have better latrine utilization while Oromia utilizes least. As a result, a random effect model was employed to estimate the pooled prevalence of latrine utilization in Ethiopia.

**Fig. 2:**
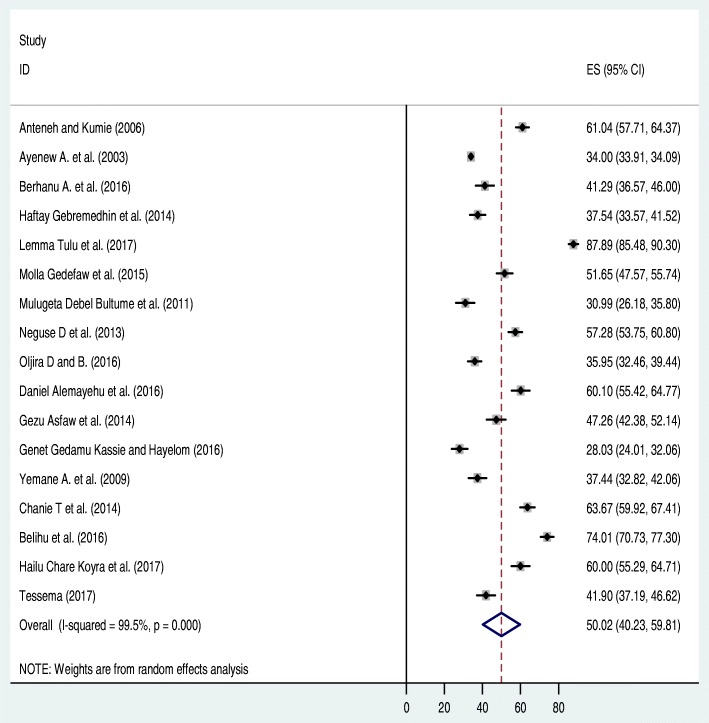
Forest plot of the pooled prevalence of latrine utilization in Ethiopia

**Fig. 3:**
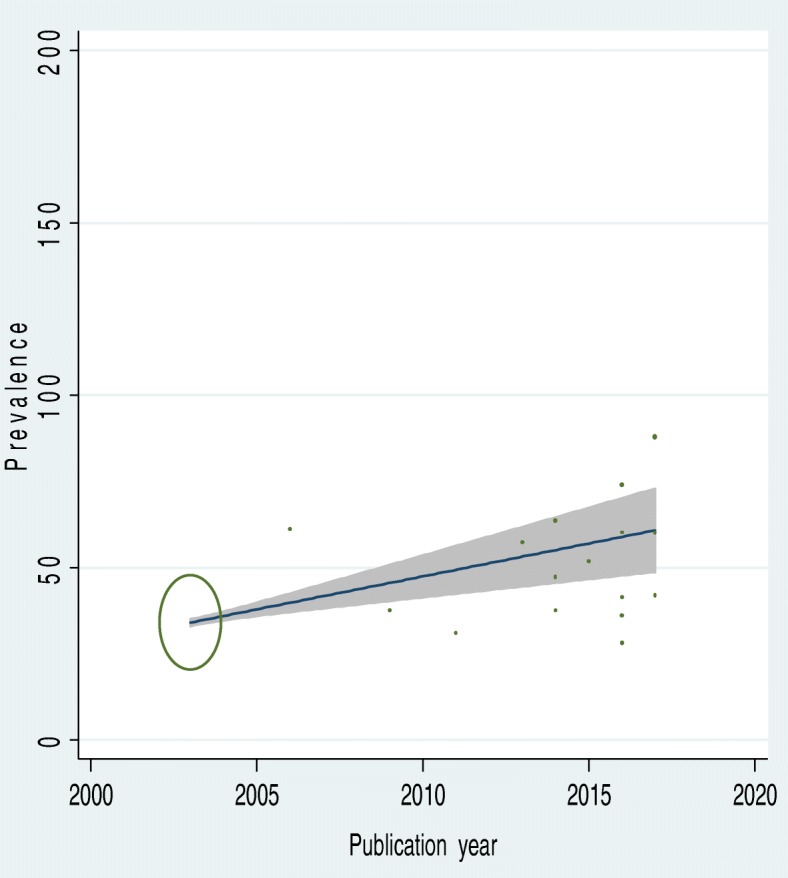
The Univariate Meta regression to identify possible source of heterogeneity by publication year

**Fig. 4:**
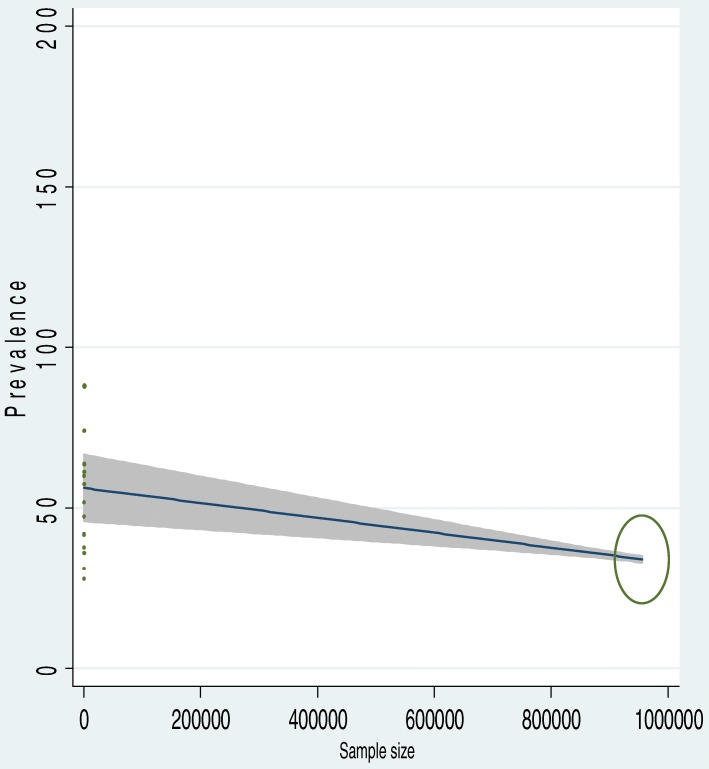
The Univariate Meta regression to identify possible source of heterogeneity by sample size

To identify the possible source of heterogeneity, different factors associated with the heterogeneity such as publication year and sample size of the study were investigated by using Univariate meta-regression models, but none of these variables were found to be statistically significant. Even though it is not statistically significant for the increments of both sample size and publication year, as sample size, increase the level of latrine utilization was showed slightly decreased, whereas the proportion showed level of latrine utilization increments as publication years does also (Table 1). Moreover, Publication bias was also assessed using Begg and Egger tests. The result of Begg and Egger tests were not statistically significant for estimating the level of latrine utilization (*p* = 0.15) and (*p* = 0.3) respectively (Figs. 2, 3, 4).

**Table 1:** Related factors with heterogeneity of the latrine utilization in the current meta-analysis (Based on Univariate Meta Regression)

Variables	Coefficient	*P*-value
Publication year	1.14	0.3
Sample size	−0.00001	0.3

### Subgroup analysis

In order to appreciate the heterogeneity of individual studies, subgroup analysis was conducted based on the region where the studies were conducted. The output of subgroup analysis revealed that, the highest latrine utilization was observed in south nation and nationalities and peoples of Ethiopia with a prevalence of 67.4% (95% CI: 50.3, 84.5) followed by Amhara with pooled latrine utilization of 50.1% (95% CI: 39.7, 62.2). Besides, subgroup analysis based on the sample size (≥500 and<500) of studies revealed that subgroup of sample size ≥500, 55.9% (95% CI: 40.0, 71.8%) revealed a higher latrine utilization than the subgroup of sample size < 500, 43.4% (95% CI: 34.9, 59.8%) (Table 2).

**Table 2:** Subgroup prevalence of latrine utilization among regions of Ethiopian country, 2017 (*n* = 17)

Variables	Characteristics	Number of studies included	Sample size	Estimate (95% CI)
By Region	Amhara	6	3350	50.1 (39.7, 62.2)
	Tigray	4	957,733	41.5 (30.9, 52.2)
	SNNPE	4	2204	67.4 (50.3, 84.5)
	Others	3	1501	36.3 (30.7, 41.9)
By sample size	≥500	9	3332	55.9 (40.0,71.8)
	< 500	8	961,456	43.4 (34.9, 59.8)
Overall		17	964,788	50.0 (40.2, 59.8)

**Fig. 5:**
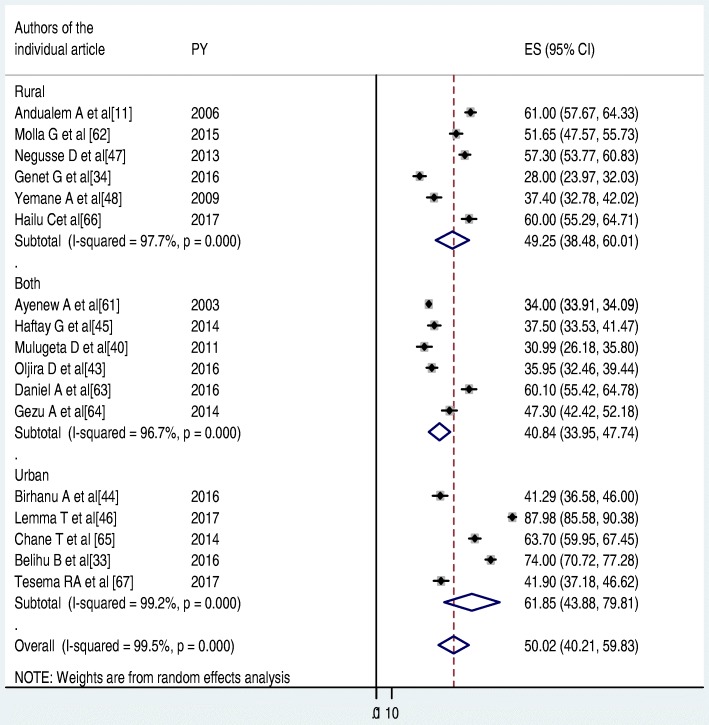
The subgroup analysis of latrine utilization status by study settings (rural, both urban and rural, and urban) in Ethiopia

Similarly, subgroup analysis was also performed between study settings (urban, rural and both). The pooled latrine utilization for study settings that means rural, both and urban were found to be 49.25(38.48, 60.01), 40.84(33.95, 47.74) and 61.85(43.88, 79.81) respectively (Fig. 5).

### The association between latrine utilization and educational status

A total of 7(41.2%) studies that fulfilled the inclusion criteria and which were considered for determining the pooled level of latrine utilization assessed the association between education and latrine utilization practice. Only one (14.3%) of the studies estimated that education has a negative association with latrine utilization. That is, respondents who are literate are less likely to utilize latrine compared with the illiterate respondents [46].

**Fig. 6:**
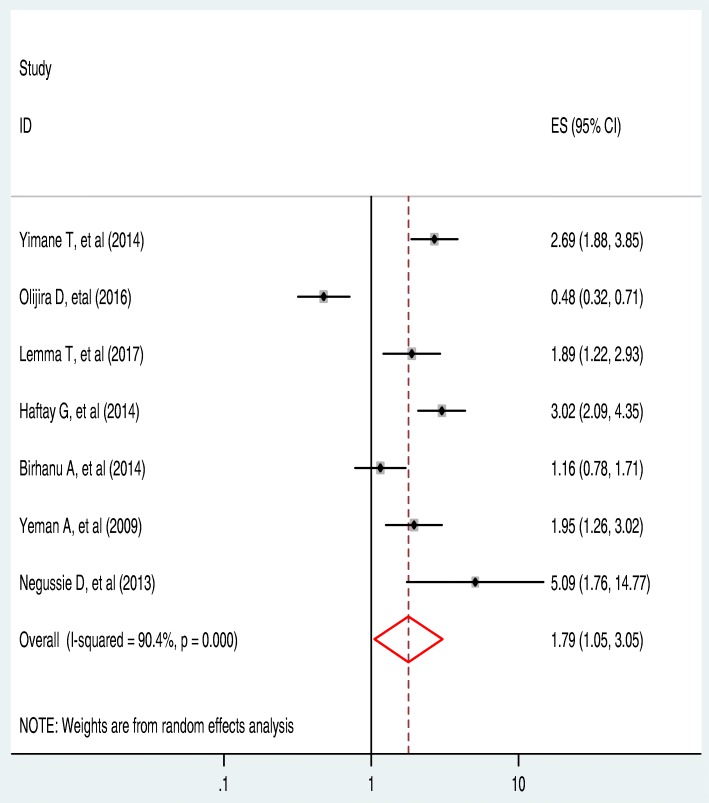
The pooled odds ratio of the association between latrine utilization and educational status in Ethiopia

The remaining 6(85.7%) of the studies reported that [44, 47–51] as people get education they use a latrine (Positive association). The heterogeneity (I^2^ = 90.4% and *P*-value < 0.001) became lower during this subgroup analysis when compared with the pooled latrine use analysis result. However, lower heterogeneity (compared with the pooled results of all 17 studies) was observed during subgroup analysis, a random effect meta-analysis model was employed to determine the association between latrine utilization and educational status of the respondents. The overall effect of educational status (as indicated in this subgroup analysis) showed that individual educational status was significantly associated with latrine utilization (OR: 1.79, 95%CI: 1.05, 3.05) (Fig. 6).

## Discussion

This systematic review and meta-analysis disclosed that the pooled level of latrine utilization in Ethiopia was 50.0% (95% CI: 40.23, 59.81%). This finding is lower than a study conducted in Ghana which revealed that 66.5% of the community had proper latrine utilization [52]. Similarly, the finding of this meta-analysis is slightly lower that a study conducted in Sub-Saharan African countries revealed that proper latrine utilization was estimated to be 63%). Likewise, the finding is much lower than a community-based study conducted in Nepal (94.3%) [53]. The possible explanation for the above-observed discrepancy between the current meta-analysis and comparable findings might be due to the difference in the Sociodemographic characteristics of the study participants. A report from other sub-Saharan African countries contained a data mostly collected from the urban population while in this study; both urban and rural settings were considered. The other possible explanation for the above variation could be due to the difference in study design.

However, the current pooled analysis of latrine utilization is higher than a the world health organization report (39%) [10]. Similarly, the current pooled latrine utilization result is slightly higher than from southern Asia countries reported by the world health organization [54] and Indian where only 47% of the respondents use latrine always [55]. The observed discrepancy could be resulted from time, study setting, sample size and socioeconomic difference among the different settings. The additional possible justification for the discrepancy might be because of half the population of in developing world lacks basic sanitation [56]. In addition, in Ethiopia, the government has been implementing different interventions to improve the level of latrine utilization (basic sanitation) for example, the implementation of health extension package since 2003. The health policy (focused on prevention of diseases and promotion of health using the provision of basic sanitation in all level of the country) of Ethiopia could be also another determinant factor for the slight improvement of the latrine utilization level in the country [57].

The pooled prevalence of latrine utilization level in Southern Nation and nationalities and People region of Ethiopia (SNNRPE) was 67.4% which is higher than the pooled prevalence of latrine utilization level than other regions of the country Ethiopia; in Amhara 50.1%, in Tigray 41.5% and others 36.3%. The possible explanations for this variation might be due to the difference in socioeconomic and sociocultural difference between the regions. The other possible explanation for this variation might be due to differences in the study period in which data collection period for all studies taken from the SNNRPE is recent than the others. On top of the above possible justification, almost half of the studies conducted at south nation nationality and people of Ethiopia were conducted in urban set-up including the capital city of the region. In addition, from this subgroup analysis, it was observed that the estimated latrine utilization level in the SNNRPE was 64.7%, which is higher than the estimated report on latrine utilization by the Min Ethiopian demographic health survey 2014 which was 54% [58] of the community use latrine. The subgroup analysis also revealed that latrine utilization was better in Amhara region next to the SNNRPE followed by Tigray. This finding was directly related with the educational development of the regions. Currently, the quality of education reported better at Tigray, Amhara region, Oromia and SNNPE, while bringing quality education on the rest regions are still challenging due to their living style. People living other than these detailed above lives a nomadic and pastoral life.

In this systematic review and meta-analysis, we performed a subgroup analysis by study settings (urban, both setting and rural). However, the finding was not statistically significant despite a slight discrepancy. Latrine utilization was found to be better in the urban setting (61.85%) as compared to rural (49.25%) and both (40.84%). Better latrine utilizations in urban setting might be due to high literate populations reside urban than the counterpart settings.

Educational level of the respondents has a significant association with latrine utilization. The finding of this study is supported by other similar study conducted on the impact of sanitation intervention on latrine coverage and uses a worldwide report that means education level has an effect on the community latrine utilization [59]. This might be due to that education has a significant influence on human behavior towards behaving health activities. Similarly as peoples’ educational status increases, their knowledge on the diseases causation, transmission and the role of human waste to the occurrence of communicable diseases increases. Therefore, to keep their health well they manage and dispose of every type of wastes (including human excreta) safely wherein properly constructed latrine. On the contrary to this study, educational status of the respondents (head of the household) has no any significant association with latrine utilization in one study conducted in Nepal [60]. This might be due to the fact that even though slightly more than half of the participates were illiterate (51.7%), the government of Nepal is committed to improving sanitation throughout the country, one priority campaign is improving latrine coverage towards attaining open defecation free areas all over the country by 2017 [17]. Despite the fact that a lot activities and strategies(like training manpower, ONE WASH, Health Extension Package and Community Lead Total Sanitation and Hygiene Behavioral Change) have been conducted in the country Ethiopia, latrine utilization was remain on half of the country vision which was 100% basic sanitation (including proper latrine utilization) [61].

Education and creating awareness is one among the 16 packages included in the health extension packages. Health extension workers employed to implement this packages provide a routine health education to improve the community awareness to increase latrine utilizations [62]. This implies that as the educational level of individual increased latrine utilization will increase.

### Limitations of the study

In this systematic review and meta-analysis, we recognized some limitations. The first concern was the use of only English language articles as inclusion criteria. The other constraint is the cross sectional nature of the included articles, which can affect the second objective due to the presence of confounders. In addition, the pooled prevalence might not represent the whole country as the included articles were only from six administrative regions.

## Conclusion

Only half of the community has had latrine utilization practice and which is lower compared with the country target 100% set to be met by 2015. This meta-analysis also showed that educational status of the community has a significant association with latrine utilization; that is, attending formal education is a positive predictor for community latrine utilization.

## Additional files

Additional file 1: Quality assessment of 19 included studies. (DOCX 17 kb)

Additional file 2: Descriptive summary of 19 studies included in the meta-analysis of the level of latrine utilizations and its association with educational status in Ethiopia. (DOCX 22 kb)

## Abbreviations

EDHS: Ethiopian Demographic and Health Survey
MDGs: Millennium Development Goals
PRISMA: Preferred Reporting of Systematic Reviews and Meta-Analysis
SNNRE: Southern Nation and Nationalities People region of Ethiopia
WASH: Water Sanitation and hygiene
