# Supplementary material for: Household latrine utilization and its association with educational status of household heads in Ethiopia: a systematic review and meta-analysis
Source: BMC Public Health. 2018 Jul 20;18:901. doi: 10.1186/s12889-018-5798-6 (PMC6053729; doi:10.1186/s12889-018-5798-6)
Supplement: Supplementary file 1 — Quality assessment of 19 included studies. (DOCX 17 kb) [file 12889_2018_5798_MOESM1_ESM.docx]

| **Articles** | **Selection 1)**  **a=b=1, c=d=0** | **Selection 2)**  **a=1, b=0** | **Selection 3)**  **a=1, b=0, c=0** | **Selection 4)**  **a=2, b=1, c=0** | **Comparability 1) a=b=1** | **Outcome 1)**  **a=b=2, c=1, d=0** | **Outcome 2)**  **a=1, b=0** | **Score** |
| --- | --- | --- | --- | --- | --- | --- | --- | --- |
| Andualem A et al | a) | a) | a) | c) | a) b) | b) | a) | 8 |
| Ayenew A et at | a) | a) | a) | a) | a)b) | c) | a) | 9 |
| Birhanu A et al | a) | a) | b) | a) | a) b) | a) | a) | 9 |
| Haftay G et al | a) | a) | a) | b) | a) b) | c) | a) | 8 |
| Lemma T et al | a) | a) | a) | a) | a) b) | b） | a) | 10 |
| Molla G et al | a) | a) | a) | a) | a) b) | b) | a) | 10 |
| Mulugeta D et al | a) | a) | a) | b) | a) b) | b) | a) | 9 |
| Sahlu C et al | a) | a） | a) | b) | a) b) | c) | a) | 8 |
| Yimam T et al | a) | a) | b） | a) | a) b) | b) | a) | 8 |
| Negusse D et al | a) | a) | a) | a) | a) b) | c) | a) | 9 |
| Oljira D et al | b) | a) | a) | a) | a) b) | b) | b) | 9 |
| Daniel A et al | a) | a) | a) | a) | a) b) | d) | a) | 8 |
| Gezu A et al | a) | a) | b) | a) | a) b) | b) | a) | 9 |
| Genet G et al | a) | a) | a) | a) | a) b) | b) | a) | 7 |
| Yemane A et al | a) | a) | b) | b) | a) b) | d) | a) | 6 |
| Chane T et al | a) | b) | a) | b) | a) b) | b) | a) | 8 |
| Belihu B et al | a) | a) | a) | c) | a) b) | a) | a) | 9 |
| Hailu Cet al | a) | a) | a) | c) | a) b) | c) | a) | 7 |
| Tesema RA et al | a) | a) | a) | c) | a) b) | b) | a) | 8 |

NB: high quality score greater than or equal to 6
