# Supplementary material for: Household latrine utilization and its association with educational status of household heads in Ethiopia: a systematic review and meta-analysis
Source: BMC Public Health. 2018 Jul 20;18:901. doi: 10.1186/s12889-018-5798-6 (PMC6053729; doi:10.1186/s12889-018-5798-6)
Supplement: Supplementary file 2 — Descriptive summary of 19 studies included in the meta-analysis of the level of latrine utilizations and its association with educational status in Ethiopia. (DOCX 22 kb) [file 12889_2018_5798_MOESM2_ESM.docx]

| Authors of the individual article | PY | Region | Study Setting | Design | Study period | Sample size | Prevalence with 95% CI |
| --- | --- | --- | --- | --- | --- | --- | --- |
| Andualem A et al[[11](#_ENREF_11)] | 2006 | Amhara | Rural | CS | June-July/ 2014 | 824 | 61.0(577, 64.4) |
| Ayenew A et al[[61](#_ENREF_61)] | 2003 | Tigray | Both | CS | April 2006-Jan 2009 | 955985 | 34 (33.91, 34.09) |
| Birhanu A et al[[44](#_ENREF_44)] | 2016 | Amhara | Urban | CS | July 2010-July 2011 | 419 | 41.29(36.57, 46) |
| Haftay G et al[[45](#_ENREF_45)] | 2014 | Tigray | Both | CS | Sep 2011- Feb 2012 | 570 | 37.5(33.57, 41.5) |
| Lemma T et al[[46](#_ENREF_46)] | 2017 | SNNPE | Urban | CS | Sep 2010 –March 2012 | 702 | 87.98(85.5, 90.3) |
| Molla G et al [[62](#_ENREF_62)] | 2015 | Amhara | Rural | CS | NR | 575 | 51.65(47.6, 55.7) |
| Mulugeta D et al[[40](#_ENREF_40)] | 2011 | Oromia | Both | CS | Sep 2103 –Sep 2014 | 355 | 30.99(26.2, 35.8) |
| Sahlu C et al [[10](#_ENREF_10), [42](#_ENREF_42)] | 2017 | Amhara | Rural | CS | April 1-10, 2016 | 768 | 98.6(97.7, 99.4) |
| Yimam T et al[[41](#_ENREF_41)] | 2013 | Amhara | Rural | CS | Nov 2009- Dec 2009 | 806 | 99.4(98.8, 99.9)) |
| Negusse D et al[[47](#_ENREF_47)] | 2013 | Tigray | Rural | CS | Feb.2014-May 2014 | 756 | 57.3(53.8, 60.8) |
| Oljira D et al[[43](#_ENREF_43)] | 2016 | Oromia | Both | CS | Feb 30 -June 2002 | 726 | 35.95(32,5, 39.4) |
| Daniel A et al[[63](#_ENREF_63)] | 2016 | Amhara | Both | CS | July15,2013-Oct.30,2013 | 421 | 60.1)55.4, 64.8) |
| Gezu A et al[[64](#_ENREF_64)] | 2014 | SNNPE | Both | CS | October 1-30/2014 | 402 | 47.3(42.4, 52.14) |
| Genet G et al[[34](#_ENREF_34)] | 2016 | Amhara | Rural | CS | January - June 2016 | 478 | 28(24.01, 32.06) |
| Yemane A et al[[48](#_ENREF_48)] | 2009 | Tigray | Rural | CS | November 2014-July2015 | 422 | 37.4(32.8, 42) |
| Chane T et al [[65](#_ENREF_65)] | 2014 | Amhara | Urban | CS | NR | 633 | 63.7(59.9, 67.4) |
| Belihu B et al[[33](#_ENREF_33)] | 2016 | SNNPE | Urban | CS | June 1-30, 2014 | 685 | 74(70.7, 77.3) |
| Hailu Cet al[[66](#_ENREF_66)] | 2017 | SNNPE | Rural | CS | February 1-30, 2013 | 415 | 60(55.3, 64.7) |
| Tesema RA et al [[67](#_ENREF_67)] | 2017 | Harar | Urban | CS | June 1-30, 2014 | 420 | 41.9(37.2, 46.6) |
